# *Plasmodium falciparum* Malaria Elicits Inflammatory Responses that Dysregulate Placental Amino Acid Transport

**DOI:** 10.1371/journal.ppat.1003153

**Published:** 2013-02-07

**Authors:** Philippe Boeuf, Elizabeth H. Aitken, Upeksha Chandrasiri, Caroline Lin Lin Chua, Bernie McInerney, Leon McQuade, Michael Duffy, Malcolm Molyneux, Graham Brown, Jocelyn Glazier, Stephen J. Rogerson

**Affiliations:** 1 The University of Melbourne, Department of Medicine (RMH), Parkville, Victoria, Australia; 2 Victorian Infectious Diseases Service, Royal Melbourne Hospital, Parkville, Victoria, Australia; 3 Australian Proteome Analysis Facility (APAF), Macquarie University, Sydney, New South Wales, Australia; 4 Malawi-Liverpool-Wellcome Trust Clinical Research Programme, College of Medicine, University of Malawi, Blantyre, Malawi; 5 School of Tropical Medicine and University of Liverpool, Liverpool, United Kingdom; 6 The Nossal Institute for Global Health, The University of Melbourne, Parkville, Victoria, Australia; 7 Maternal and Fetal Health Research Centre, University of Manchester, St. Mary's Hospital, Manchester, United Kingdom; Case Western Reserve University, United States of America

## Abstract

Placental malaria (PM) can lead to poor neonatal outcomes, including low birthweight due to fetal growth restriction (FGR), especially when associated with local inflammation (intervillositis or IV). The pathogenesis of PM-associated FGR is largely unknown, but in idiopathic FGR, impaired transplacental amino acid transport, especially through the system A group of amino acid transporters, has been implicated. We hypothesized that PM-associated FGR could result from impairment of transplacental amino acid transport triggered by IV. In a cohort of Malawian women and their infants, the expression and activity of system A (measured by Na^+^-dependent ^14^C-MeAIB uptake) were reduced in PM, especially when associated with IV, compared to uninfected placentas. In an *in vitro* model of PM with IV, placental cells exposed to monocyte/infected erythrocytes conditioned medium showed decreased system A activity. Amino acid concentrations analyzed by reversed phase ultra performance liquid chromatography in paired maternal and cord plasmas revealed specific alterations of amino acid transport by PM, especially with IV. Overall, our data suggest that the fetoplacental unit responds to PM by altering its placental amino acid transport to maintain adequate fetal growth. However, IV more profoundly compromises placental amino acid transport function, leading to FGR. Our study offers the first pathogenetic explanation for FGR in PM.

## Author Summary

Malaria infection during pregnancy can cause fetal growth restriction and low birthweight associated with high infant mortality and morbidity rates. The pathogenesis of fetal growth restriction in placental malaria is largely unknown, but in other pathological pregnancies, impaired transplacental amino acid transport has been implicated. In a cohort of Malawian women and their infants, we found that placental malaria, especially when associated with local inflammation, was associated with decreased expression and activity of an important group of amino acid placental transporters. Using an *in vitro* model of placental malaria with local inflammation, we discovered that maternal monocyte products could impair the activity of amino acid transporters on placental cells. Amino acid concentrations in paired maternal and cord plasmas revealed specific alterations of amino acid transport by placental malaria, especially with local inflammation. Overall, our data suggest that, more than malaria infection *per se*, the local inflammation it triggers compromises placental amino acid transport function, leading to fetal growth restriction. Greater understanding of the mechanisms involved, combined with interventions to improve fetal growth in malaria, are important priorities in areas of the world where the co-existence of malaria and maternal malnutrition threatens the health and lives of millions of young babies.

## Introduction

Pregnant women living in malaria endemic regions are highly susceptible to malaria, especially in first pregnancies [1], [2]. Malaria in pregnancy is characterized by placental malaria (PM), the selective accumulation of *Plasmodium-falciparum* infected erythrocytes (IE) in the maternal intervillous blood space of the placenta, in direct contact with the nutrient-transporting epithelium, the syncytiotrophoblast. When placental malarial infection is poorly controlled, chemokine release results in the recruitment of maternal immune cells, predominantly monocytes, to the intervillous blood spaces [3]. The resultant inflammation is termed intervillositis (IV) [4]. In comparison to PM without local inflammation, PM with IV is associated with significant decreases in birthweight and an increased prevalence of low birthweight (LBW) deliveries, primarily due to fetal growth restriction (FGR) [1], [2], [5], [6].

Recent studies have begun to shed light on the pathogenetic mechanisms linking PM and FGR (reviewed in [7]). Inadequate maternal nutrition and placental insufficiency have been proposed. In Congolese women studied by serial ultrasound examinations, FGR associated with PM was 2–8 times more common in undernourished than in well-nourished mothers [8]. The same undernourished mothers with PM had increased uterine artery resistance (Griffin *et al.* submitted), which is associated with placental insufficiency. A decreased fetal/placental weight ratio is one manifestation of placental insufficiency found in primigravid women with PM [9].

It has previously been suggested [9], [10] that FGR and LBW associated with PM could be caused by impaired capacity of the placenta to transport maternal nutrients, especially amino acids, to the growing fetus. Although this postulate has never been formally tested, it is supported by observations in idiopathic FGR showing that the activities of various placental nutrient transporters are selectively altered [11], [12]. Among the nutrient transporters affected is system A, a group of Na^+^-dependent neutral amino acid transporters that actively transfer small, neutral amino acids and thereby enables the establishment of high intracellular amino acid concentrations, which are then used to exchange for extracellular essential amino acids via system L [13], [14].

In the placenta, system A activity is mediated by three Na^+^-dependent neutral amino acid transporter (SNAT) isoforms belonging to the *SLC38* gene family; SNAT1 (*SLC38A1*), SNAT2 (*SLC38A2*) and SNAT4 (*SLC38A4*). All isoforms are expressed on the microvillous plasma membrane (MVM) of the human syncytiotrophoblast [15]. A reduced system A amino acid transporter activity in MVM has been consistently observed in placentas of pregnancies associated with FGR [16]–[18], and the reduction in system A activity in MVM correlates well with the severity of FGR [19]. Further, various animal studies have suggested that reduced system A activity may be causally related to the etiology of FGR [20]–[22].

Pro-inflammatory cytokines produced by monocytes have been shown to decrease system A activity. IL-1β reduces system A activity in trophoblast cells [23] and acute exposure to TNF-α resulted in diminished maternofetal transfer of a system A analogue in a rat model of FGR [24]. These cytokines have been associated with LBW in PM, especially when associated with IV [25]–[27], and their production could be caused by PM, either through activation of monocytes by IE [28] or by direct effects of IE on syncytiotrophoblast leading to secretion of cytokines and chemokines [29], [30]. This suggests a link between PM, IV and altered placental amino acid transport, with impacts on fetal growth and development.

In the current study, we hypothesized that the release of soluble mediators triggered by IV associated with PM impairs placental transport of amino acids across the syncytiotrophoblast, contributing to the pathogenesis of FGR and LBW. We found that PM, especially with IV, was associated with decreased placental amino acid uptake and dysregulated maternofetal amino acid balance, likely to alter the transfer of amino acids to the fetus, and to contribute to the pathogenesis of PM-associated FGR.

## Results

### Participants' characteristics

Characteristics of the individuals who participated in the various aspects of the study are summarized in Table 1.

**Table 1:** Participants' characteristics.

	Uninfected	Malaria alone	Malaria with intervillositis	Kruskal-Wallis' test
***SLC38A1*** ** AND ** ***SLC38A2*** ** mRNA expression**				
*N*	21	11	21	
*Gestational Age*	40	39	39	0.31
*(in weeks)*	(39–40)	(35–41)	(36–41)	
*Age*	20	23	21	0.085
*(in years)*	(16–29)	(18–29)	(15–27)	
*Gravidity*	1	3	1	0.081
	(1–3)	(1–4)	(1–5)	
*Maternal Hb*	11	10.8	11	0.85
*(in g/dL)*	(8.7–15.2)	(7.6–12.9)	(6–14)	
*Fetal weight*	3100	3135	2925	0.039^A^
*(in grams)*	(2500–4200)	(2500–3700)	(2000–3650)	
*Maternal weight at*	57.5	56	55	0.91
*enrolment (in kg)*	(50–100)	(44–69)	(44–75)	
**MeAIB uptake**				
*N*	18	14	21	
*Gestational Age*	40	40.5	39	0.073
*(in weeks)*	(38–40)	(35–50)	(36–41)	
*Age*	18	18	18	0.63
*(in years)*	(16–23)	(16–33)	(16–24)	
*Gravidity*	1	1	1	0.43
	(1–3)	(1–3)	(1–2)	
*Maternal Hb*	12.8	11.8	10.9	0.038^B^
*(in g/dL)*	(9–15.1)	(4–13.8)	(3.3–14.1)	
*Fetal weight*	3050	2925	2800	0.15^C^
*(in grams)*	(2600–3800)	(2200–3200)	(2100–4100)	
*Maternal weight at*	57.5	58	52	0.048^D^
*enrolment (in kg)*	(45–69)	(56–60)	(46–60)	
**Free Amino Acid Analysis**				
*N*	31	11	28	
*Gestational Age*	40	40	38	0.24
*(in weeks)*	(36–42)	(35–41)	(36–42)	
*Age*	18	19	18.5	0.75
*(in years)*	(16–23)	(16–25)	(16–24)	
*Gravidity*	1	1	1	1^E^
	(1–1)	(1–1)	(1–1)	
*Maternal Hb*	13	11.8	10.75	0.0004^B^
*(in g/dL)*	(9–16.1)	(10–15.8)	(6.7–13.4)	
*Fetal weight*	3000	2900	2800	0.28
*(in grams)*	(1990–3800)	(2200–3600)	(2000–4100)	
*Placental weight*	500	500	510	0.90
*(in grams)*	(340–650)	(400–620)	(300–730)	
*Fetal to placental*	6	5.85	5.41	0.11^F^
*weight ratio*	(4.14–8.23)	(4.49–7.25)	(3.81–7.67)	
*Maternal weight at*	55	54	53	0.75
*enrolment (in kg)*	(42–70)	(48–60)	(45–60)	

Data are shown as median (interquartile range).

A Infants born of mothers with PM and IV were lighter than those born of mothers in the two other groups.

B Mothers with PM and IV had lower Hb concentration than uninfected mothers or mothers with PM without IV. The maternal Hb concentration in the latter two groups did not differ.

C There was a trend for a decreased foetal weight across groups (p = 0.053).

D Mothers with PM with IV were lighter than mothers with PM without IV and than uninfected mothers. Maternal weight at enrolment did not differ between uninfected mothers and mothers with PM without IV.

E Only primigravidae were recruited in this study.

F PM with IV cases had lower foetal/placental weight ratio than uninfected controls (p = 0.036).

### *SLC38A1* and *SLC38A2* transcript levels are altered in placental malaria with intervillositis

**Figure 1:**
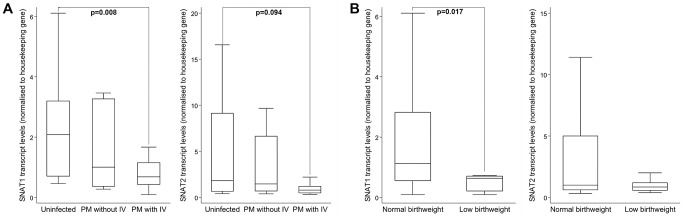
*SLC38A1* and *SLC38A2* mRNA expression in syncytiotrophoblast and relationship to birthweight. Expression of mRNA for the amino acid transporters *SLC38A1* and *SLC38A2* was quantified in the laser-captured syncytiotrophoblast of placental biopsies by real time qPCR and expressed normalized to the housekeeping gene YWHAZ. **A.** *SLC38A1* mRNA expression in placental malaria (PM) with intervillositis (IV; n = 21) was lower than in uninfected placentas (n = 21; p = 0.008). *SLC38A1* transcript levels in placental malaria without intervillositis (n = 11) were intermediate and similar to that in uninfected placentas (p = 0.46) and to that in infected placentas with IV (p = 0.23). A similar profile was observed for *SLC38A2* mRNA expression comparing expression in infected placentas with IV and uninfected placentas. **B.** Placentas from infants born with a low birthweight (regardless of infection status; n = 7) had lower *SLC38A1* (p = 0.017) but similar *SLC38A2* (p = 0.39) transcript levels compared to those born with a normal birthweight (n = 46). Data shown were obtained in an experiment run in triplicate.

*SLC38A1* transcript levels were reduced (p = 0.008) in the syncytiotrophoblast of infected placentas with IV compared to that of uninfected placentas, while levels in syncytiotrophoblast of infected placentas without IV were intermediate. A similar trend was observed for *SLC38A2* transcript levels (Fig. 1A). *SLC38A1* (p = 0.017) but not *SLC38A2* (p = 0.39) transcript levels were lower in the syncytiotrophoblast of placentas of LBW infants compared to normal birthweight infants (Fig. 1B).

### Amino acid uptake is reduced in placental malaria with intervillositis

**Figure 2:**
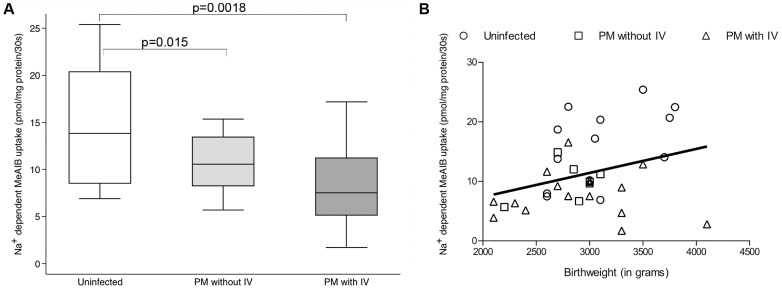
Amino acid uptake and relationship to placental malaria and birthweight. MVM was purified from uninfected placentas (n = 18) and infected placentas without (n = 14) or with (n = 22) intervillositis (IV) and Na^+^-dependent MeAIB uptake was quantified in duplicate. **A.** Na^+^-dependent MeAIB uptake by MVM vesicles from infected placentas both with or without IV was significantly lower compared to that of uninfected placentas. Na^+^-dependent MeAIB uptake by MVM of infected placentas with or without IV was not significantly different (p = 0.65). Data are represented as median (horizontal line), interquartile range (box) and 5th/95^th^ centiles (whiskers). **B.** Correlation between Na^+^-dependent MeAIB uptake by MVM vesicles and birthweight (n = 49; Rho = 0.26; p = 0.07). Uninfected women are represented by ○, placental malaria (PM) without IV by □ and PM with IV by Δ.

Figure 2A reveals that Na^+^-dependent MeAIB uptake by MVM vesicles from infected placentas either with or without IV was lower (p≤0.015) than uptake by vesicles from uninfected placentas. Na^+^-dependent MeAIB uptake was similar between groups with PM (p = 0.65). Birthweight was positively associated with Na^+^-dependent MeAIB uptake by MVM vesicles from all placentas (Rho = 0.26, p = 0.07; Fig. 2B).

### IL-1β concentration is elevated in placental malaria with intervillositis

In response to *P. falciparum* infection, monocytes elicit a pro-inflammatory response including the secretion of IL-1β [28]. IL-1β has been previously reported to decrease Na^+^-dependent MeAIB uptake by placental trophoblast cells [23]. We therefore investigated whether PM with IV was associated with increased IL-1β concentration and if conditioned medium from a monocyte/IE co-culture could impair Na^+^-dependent MeAIB uptake.

**Figure 3:**
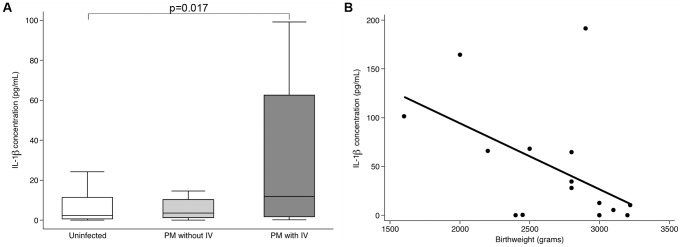
IL-1β placental plasma concentration and relationship to birthweight. **A.** IL-1β concentration in plasma sampled from blood of infected placentas with intervillositis (IV; n = 16) was higher (p = 0.017) than that of uninfected placentas (n = 31). IL-1β concentration in infected placentas without IV (n = 16) was intermediate and similar to concentrations in infected placentas with IV (p = 0.1) and to concentrations in uninfected placentas (p = 0.39). Data are represented as median (horizontal line), interquartile range (box) and 5th/95^th^ centiles (whiskers). The ELISA was performed in duplicate. **B.** IL-1β concentration in plasma sampled from blood of infected placentas with intervillositis showed a negative correlation with birthweight (n = 16; Rho = −0.52; p = 0.04).

IL-1β plasma concentration in maternal blood harvested from placentas with PM and IV was higher (p = 0.017) compared to uninfected controls, and comparable (p = 0.1) to the PM without IV group (Fig. 3A). Within the group of PM with IV, IL-1β concentration was negatively correlated with birthweight (Rho = −0.52; p = 0.04; Fig. 3B).

### IE stimulate monocytes to secrete factors that decrease amino acid uptake by BeWo cells

Because IL-1β is produced by monocytes in response to IE [28] and because Na^+^-dependent MeAIB uptake by MVM vesicles was lowest in PM with IV (Fig. 2A), we speculated that system A activity impairment could be attributable to products generated by monocytes in response to IE.

Medium collected from a monocyte/IE co-culture was used to mimic the intervillous space milieu in cases of PM with IV. This medium was applied to human placental choriocarcinoma BeWo cells to investigate its effect on Na^+^-dependent MeAIB uptake. Cell viability was monitored in all subsequent experiments and was unaffected by any of the treatments (data not shown).

**Figure 4:**
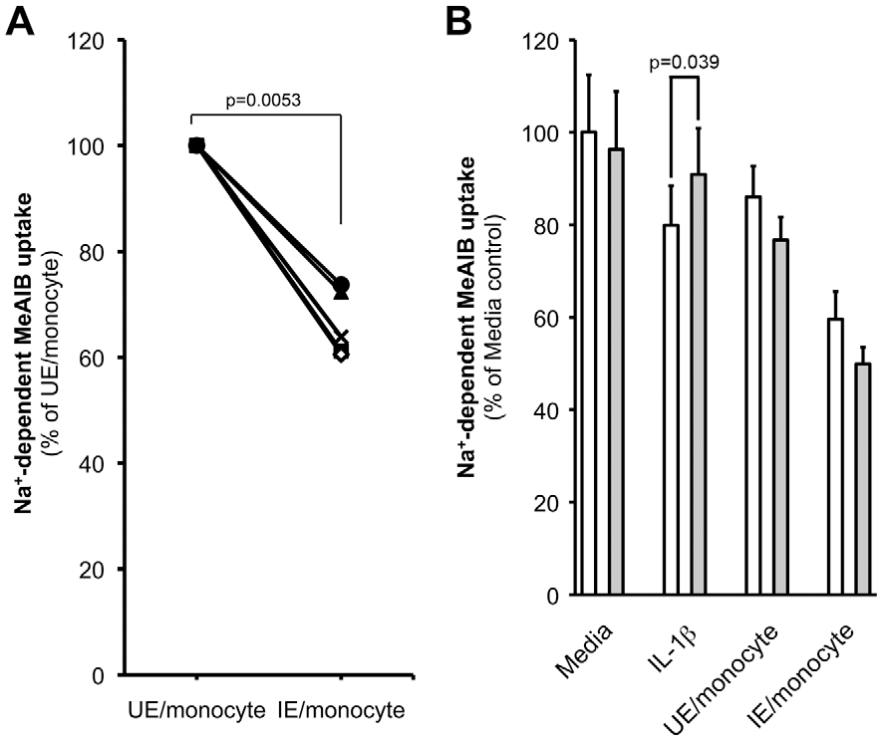
*In vitro* model of amino acid uptake by placental cells. **A.** BeWo cells incubated for 16 h with the conditioned medium from an IE/monocyte co-culture showed a marked decrease in Na^+^-dependent MeAIB uptake relative to cells incubated with an uninfected erythrocyte (UE)/monocyte conditioned medium (n = 5 independent donors in triplicate). **B.** BeWo cells were treated for 16 h with either recombinant IL-1β (5 ng/mL) or conditioned media from UE/monocyte or IE/monocyte co-culture in the presence (grey bars) or absence (open bars) of IL-1β blocking antibody (BA) (1.25 µg/mL) and Na^+^-dependent MeAIB uptake measured. Data are expressed as mean + SD (n = 2 independent experiments in triplicate). Effect of the BA was tested using a one-sided two-sample T-test.

An inhibition of Na^+^-dependent MeAIB uptake by BeWo cells when exposed to monocyte/IE co-culture conditioned media was consistently observed (Fig. 4A). An IL-1β blocking antibody was used to investigate the role of IL-1β in mediating this effect (Fig. 4B). At the concentration used, the blocking antibody was effective in abolishing recombinant IL-1β-mediated reduction in Na^+^-dependent MeAIB uptake. In contrast, addition of IL-1β blocking antibody had no effect on the inhibition of Na^+^-dependent MeAIB uptake observed with monocyte/IE conditioned media. This indicated that IL-1β was not a major factor in the reduction in Na^+^-dependent MeAIB uptake observed with monocyte/IE conditioned media.

Treatment of BeWo cells with uninfected erythrocytes (UE) or with lysed or intact IE that had or had not been opsonized by human Ig did not alter Na^+^-dependent MeAIB uptake by BeWo cells compared to media control (p≥0.12; data not shown). This suggests that the reduction in Na^+^-dependent MeAIB uptake by BeWo cells relies on monocytes' response to IE more than effects of IE *per se*.

### Placental malaria selectively alters fetal-maternal amino acid interrelationships

We next investigated the potential effect of altered system A activity, indicated by the reduced Na^+^-dependent MeAIB uptake observed in PM with IV, on fetal amino acid levels by measuring free amino acid concentration in paired maternal and cord plasma samples (Table 2).

**Table 2:** Maternal and cord amino acid concentration.

	Uninfected (n = 31)		PM without IV (n = 11)		PM with IV (n = 28)	
	Maternal levels	Cord levels	Maternal levels	Cord levels	Maternal levels	Cord levels
	(µM)	(µM)	(µM)	(µM)	(µM)	(µM)
**Non essential AA**						
Alanine	387.19^BBB^	584.66^BB^	319.62	510.25	295.14	464.59
	(301.2–438.72)	(458.74–685.69)	(250.66–354.37)	(478.85–754.49)	(213.38–358.48)	(396.84–557.17)
Arginine	23.9	48.39	24.55	48.3	28.28	47.79
	(19.83–39.85)	(33.48–69.61)	(14.87–26.59)	(30.83–79.52)	(20.46–38.63)	(38.15–59.75)
Asparagine	25.66^AA^	51.01^A^	29.17	55.78	24.97	52.01
	(21.34–28.84)	(44.4–57.45)	(23.85–33.47)	(42.41–97.69)	(20.26–33.09)	(45.07–68.58)
Aspartic acid	7.82^AA,BB^	30.74^AA^	9.97	35.46^C^	9.32	29.05
	(4.91–8.98)	(15.32–37.41)	(9.26–12.12)	(17.74–128.17)	(7.89–13.3)	(13.91–54.83)
Cystine	5.8^AA^	24.05	9.69^C^	24.38	6.87	21.07
	(4.35–8.08)	(18.49–34.12)	(8.15–14.99)	(16.33–40.88)	(4.33–9.53)	(17.02–27.61)
Glutamic acid	57.23	180.16^AA^	63.07	253.06^C^	47.75	153.32
	(44.86–78.64)	(126.76–233.05)	(46.3–79.45)	(116.1–603.27)	(41.56–72.26)	(113.26–243.11)
Glutamine	373.47	587.38	400.77	529.58	376.39	551.4
	(314.88–452.59)	(515.16–663.18)	(340.06–442.36)	(500–754.6)	(304.86–468.22)	(486.81–686.83)
Glycine	130	264.17^A^	125.43	313.28	118.76	283.75
	(98.37–148.81)	(240.36–328.57)	(107.69–143.34)	(236.6–399.44)	(96.48–135.31)	(233.35–350.15)
Proline	136.16	200.91	146.15	236.93	150.53	218.55
	(116.3–160.3)	(178.81–230.77)	(132.4–189.45)	(169.67–269.92)	(122.2–174.31)	(184.3–276.45)
Serine	72.81	139.12	77.09	135.57	75.53	128.23
	(60.88–87.44)	(123.79–163.95)	(63.16–89.33)	(117.59–217.13)	(62.53–87.69)	(118.49–160.1)
Tyrosine	36.56	78.75	36.8	73.05	35.23	70.84
	(32.92–43.1)	(68.12–86.43)	(30.8–44)	(65.96–96.54)	(30.34–42.56)	(59.06–85.63)
**Essential AA**						
Histidine	58.22	93.72	60.28	83.3	57.52	94.69
	(48.19–66.74)	(85.42–102.77)	(56.03–69.09)	(80.66–108.18)	(47.26–68.38)	(82.02–105.39)
Isoleucine	31.78	61.57	34.46	77.1	34.22	65.33
	(27.11–36.4)	(54.66–75.37)	(29.19–39.11)	(50.01–83.8)	(26.8–42.53)	(51.87–78.01)
Leucine	62.19^BB^	128.19^AA,BB^	66.15	145.77	78.64	143.38
	(55.99–78.88)	(109.2–149.33)	(62.36–81.59)	(99.8–209.07)	(60.01–93.25)	(106.89–179.13)
Lysine	74.96	242.63	78.24	232.63	88	266.57
	(64.46–92.91)	(210.63–281.02)	(61.61–117.25)	(183.25–294.91)	(69.21–114.05)	(186.11–311.82)
Methionine	18.14	34.73^B^	16.45	36.6^C^	18.55	30.67
	(14.56–23)	(29.37–38.73)	(14.73–23.92)	(30.28–41.8)	(14.31–22.05)	(26.58–37.47)
Phenylalanine	37.78^BBB^	83.98^AA,BBB^	39.42	91.58	47.72	92.39
	(35.11–44.69)	(72.29–89.41)	(35.72–53.66)	(77.14–122.06)	(39.37–59.26)	(79.22–115.6)
Threonine	93.89	176.51	107.22	176.59	101.9	180.12
	(73.61–111.96)	(147.92–198.51)	(88.14–121)	(155.41–221.22)	(78.83–117.47)	(146.41–207.05)
Tryptophan	4.13^AAA^	5.53^AAA^	6.47	9.66^CCC^	5.32	6.08
	(2.81–6.16)	(4.09–7.72)	(3.81–12.78)	(6.75–15.51)	(3.78–8.1)	(4.21–7.65)
Valine	93.51^BBB^	194.54^BBB^	102.62	232.04	114.8	217.62
	(84.33–101.04)	(168.31–216.49)	(80.17–127.46)	(157.56–234.44)	(93.84–136.63)	(186.64–261.6)
**Total AA**	1744.68	3263.84^A^	1741.41	3353.38	1806.95	3128.61
	(1478.9–1917.1)	(2872.7–3625)	(1566.2–2043.2)	(2692.9–4092.5)	(1441.8–1965.5)	(2828.7–4026.6)

Free amino acid concentration was quantified (in duplicate) in paired maternal and cord blood samples and expressed as median (interquartile range). PM: Placental malaria; IV: intervillositis. Key to statistical analysis:

A = Uninfected versus PM without IV;

B = Uninfected versus PM with IV;

C = PM without IV versus PM with IV. One symbol = 0.05<p≤0.1; two symbols = 0.01<p≤0.05; three symbols = p≤0.01.

#### Maternal concentrations

In mothers with PM without IV, plasma concentration of Asn, Asp, Cys and Trp was higher (p≤0.037) than in uninfected mothers. Plasma concentration of Asp, Leu, Phe and Val was higher (p≤0.022) and Ala concentration lower (p = 0.003) in women with PM and IV than in uninfected mothers.

#### Fetal concentrations

In the group of PM without IV, cord concentrations of certain amino acids (Asn, Gly, Leu, Phe and Trp: p≤0.081), especially Asp (p = 0.028) and Glu (p = 0.026) were increased compared to babies of uninfected women (all other amino acid cord concentrations were similar between the two groups; p≥0.19). In contrast, when PM was associated with IV, cord concentrations of most amino acids were similar to, or lower than (p≤0.093; Ala and Met), those of the uninfected group, with the exceptions of Leu, Phe and Val for which cord concentrations were higher (p≤0.046) in the PM with IV group compared to uninfected controls. As noted above, maternal concentrations for these three amino acids were also higher in PM with IV, and given the tight correlations between cord and maternal concentrations of these amino acids (p≤0.02; Rho≥0.33) in this group, the elevated cord concentration could be driven by the high maternal concentration. There was no correlation between cord and maternal concentrations in the uninfected group for these amino acids (p≥0.32). Overall, these data suggest that PM alone and PM with IV are each associated with altered concentrations of particular amino acids in cord blood, suggesting specificity of response.

#### Fetal-maternal concentration ratio

Fetal cord concentrations were higher than maternal concentrations (p≤0.05) for all amino acids in all groups except for Trp. Uninfected placentas were able to concentrate Trp (Median = 1.27; IQR 1.01–1.63; p = 0.0009) whereas placentas with PM with IV could not (Median = 1.24; IQR 0.84–1.62; p = 0.18). Placentas with PM without IV showed an intermediate phenotype (p = 0.065).

### Placental malaria, placental insufficiency and amino acid transport

A low fetal/placental weight ratio is a marker of placental insufficiency and has been associated with malaria [9], [31], [32] and PM with IV cases had lower fetal/placental weight ratio than uninfected controls (p = 0.036). For a number of amino acids, cord concentration was positively correlated with fetal/placental weight ratio, either among all PM cases or for those with IV (Table 3). Among babies with PM and IV, cord concentrations of several neutral, branched chain amino acids, transported by system L [13] were positively associated with fetal/placental weight ratio. This suggests that intervillositis may lead to placental insufficiency in part through impaired transplacental amino acid transport. There was no positive correlation between cord concentration of amino acids and birthweight (p≥0.29).

**Table 3:** Correlations between fetal/placental weight ratio and amino acid concentration in PM samples.

	Uninfected controls		Placental malaria (with or without intervillositis)		Placental malaria with intervillositis	
	Maternal levels	Cord levels	Maternal levels	Cord levels	Maternal levels	Cord levels
**Non essential AA**						
Alanine			0.31+	0.37++		
Arginine				0.46+++		
Asparagine				0.34++		
Aspartic acid						
Cystine						
Glutamic acid						
Glutamine				0.33++		
Glycine						
Proline						
Serine				0.4++		
Tyrosine						0.41++
**Essential AA**						
Histidine		0.81+++		0.37++		
Isoleucine				0.37++		0.41++
Leucine						0.38++
Lysine		0.55+		0.32++		
Methionine				0.33++		
Phenylalanine						
Threonine						
Tryptophan				0.32++		0.39++
Valine						
**Total AA**				0.37++		0.43++

Correlations (Spearman's rank test) between either cord or maternal amino acid concentration or their ratio and fetal/placental weight ratio (a marker of placental insufficiency) are summarized. Key to statistical analysis:

+ = 0.05<p≤0.1;

++ = 0.01<p≤0.05;

+++ = p≤0.01; all with a Rho≥0.3.

## Discussion

Understanding the pathogenesis of PM-associated FGR is critical to the design of novel interventions to decrease its burden [33]. In this study, we identified an impaired placental amino acid uptake and dysregulated maternofetal amino acid balance in PM, especially with IV, providing a pathogenic mechanism for PM-associated FGR through altered transfer of amino acids to the fetus.

The activities of various transport mechanisms in the plasma membranes of the syncytiotrophoblast are dysregulated in idiopathic FGR [34], [35]. In particular, the activity of system A amino acid transporters has often been reported to be downregulated in FGR [17]–[19], and animal studies demonstrate that a reduction in system A activity precedes development of FGR [22].

We observed reduced transcription of the system A transporters *SLC38A1* (SNAT1), and, to a lesser extent *SLC38A2* (SNAT2), within the syncytiotrophoblast in PM with IV, which is compatible with the reduction we observed in system A activity, and consistent with the involvement of these two SNAT subtypes in the downregulation of system A activity associated with PM with IV. Previous studies of idiopathic FGR have not demonstrated altered *SLC38A1* or *SLC38A2* transcription in whole placental lysates [36], whereas here we have specifically measured *SLC38A1* and *SLC38A2* transcript levels from the syncytiotrophoblast. Our data suggest that in cases of PM with IV, the syncytiotrophoblast responds to infection and inflammation by down-regulating the transcription of these SNAT isoforms. As activity of SNATs is partly regulated at the level of transcription [37], this suggests that PM with IV may decrease SNAT-mediated placental amino acid transport.

To investigate this further, we studied system A activity *ex vivo*. System A activity was decreased in PM alone, and to a greater extent in PM with IV. The relationships observed between PM or birthweight with system A activity and SNAT transcript levels suggest that system A makes important contributions to fetal growth, and that these contributions are compromised by PM, especially PM with IV.

To understand how PM, especially with IV, might impair placental amino acid transport we developed an *in vitro* model to examine trophoblast cell responses to factors present in the placental intervillous blood space. Regardless of the way they were presented to placental cells, IE alone did not induce a significant decrease in system A-mediated MeAIB uptake. This is in accord with our *ex vivo* data, and with clinical observations that PM without IV is not associated with FGR [6]. In contrast, conditioned media from monocyte-IE co-cultures, which mimic the intervillous milieu in PM with IV, significantly reduced MeAIB uptake by trophoblast cells, indicating that monocytes participated in eliciting this response. Our evidence suggests that IE activate monocytes to release factors that inhibit system A activity.

A number of factors have been shown to modulate system A activity in placental cells or BeWo layers including cytokines such as IL-1β, IL-6 and TNF-α [23], [38] which are increased in PM [25]–[27] and produced by monocytes in response to IE [28]. Despite IL-1β concentrations being raised in maternal blood of the intervillous space in PM with IV and negatively correlating with birthweight in this group, the decreased Na^+^-dependent MeAIB uptake by BeWo cells in our *in vitro* model was not substantially mediated by IL-1β, as illustrated by the inability of blocking antibody to IL-1 β to counteract the inhibitory effect of monocyte-IE co-culture supernatants; the mediator(s) responsible are at present unknown and could either be a factor(s) consumed by malaria-stimulated monocytes or a factor(s) secreted by these cells.

The cause of the decreased amino acid uptake observed *ex vivo* in MVM from women with PM and IV is not known. As discussed above, it may be mediated by monocyte-derived factors, but these remain to be conclusively identified. Hormones that stimulate system A-mediated amino acid uptake including IGFs [39] and leptin [40] are decreased in PM [41], [42], and these may contribute in part to the decreased system A activity demonstrated in patient samples. *In vitro*, supernatants from co-cultures (rather than monocytes themselves) inhibit amino acid uptake, suggesting that local depletion of available amino acids by activated monocytes is not a significant contributing factor.

We next assessed whether the observed decrease in system A activity in PM, or effects of PM on other amino acid transport systems, resulted in altered amino acid concentrations in maternal and cord blood. In normal pregnancy, delivery of some amino acids, particularly essential amino acids, is only just sufficient to meet fetal requirements [43], [44]. In pregnancies compromised by severe FGR, maternofetal transfer of amino acids may be reduced [45], [46]. In PM without IV, maternal concentration of a number of amino acids (Asn, Asp, Cys and Trp) was increased compared to uninfected controls, possibly due to reduced uptake of these amino acids by the syncytiotrophoblast, resulting in increased maternal concentrations. In PM with IV, maternal concentration of all amino acids except Ala was either unchanged or elevated compared to uninfected controls, consistent with observations in idiopathic FGR [46]. Cord Ala levels were also lower compared to uninfected controls and positively correlated with maternal levels in PM with IV (Rho = 0.56; p = 0.002). Thus, malaria-related inadequate maternal concentrations of amino acids were not responsible for changes in fetal amino acid concentrations.

We did not see widespread decreases in cord amino acid concentration in the group with PM and IV, as have been described in idiopathic FGR [19], [45]. This lack of widespread impact of PM on cord amino acid concentration could be explained by the degree of severity of the FGR in our study compared to the idiopathic FGR studies. In the latter, birthweight was dramatically decreased, by ∼600 g to ∼1550 g. In contrast, in our cohort, birthweight of control infants was relatively low, and birthweight only differed by ∼200 g between control infants and those with PM and IV (in keeping with larger epidemiological studies in this population [5], [27]). In resource-poor settings such as Malawi, obstetric care is limited, and women with at risk pregnancies due to highly compromised placental function and severe FGR may not be identified for intensive management, but may instead experience pregnancy loss. Malaria is a common cause of stillbirth and miscarriage in such settings [47], [48], and our study design may have resulted in malaria-affected pregnancies with severe FGR being under-represented in our cohorts. Longitudinal studies of at-risk pregnancies may be useful in quantifying the risks and manifestations of severe FGR in malaria-affected pregnancies further.

Differences in cord blood amino acid concentrations between groups suggest that placental transport and/or metabolism of a number of amino acids is altered in PM. In the PM without IV group, the neutral and anionic classes of amino acids were most notably affected, suggesting a selective effect on placental handling of these amino acids. In infected women without IV but not in the group with PM with IV, there was a particularly striking increase in the fetal concentration of the anionic amino acids Asp and Glu, which are taken up into the placenta from the maternal and fetal circulations respectively by system X_AG_^−^. The physiological significance of this is unclear at present and it could suggest that IV restored placental transport of these amino acids. However, it is known that Glu uptake from the fetal circulation plays a crucial role in fetoplacental Glu-Gln cycling and may also serve to protect the fetus against Glu neurotoxicity [49]. The similar trend observed for Asn, Gly, Met (system A substrates) and both Met and Leu (system L substrates) also implicates these systems as being affected differentially in PM without IV as against PM with IV. Whether this reflects altered amino acid transport and/or placental amino acid utilization or production has yet to be established. The decrease in system A activity could also indirectly impair the activity of other systems that depend on the gradient of amino acid concentration established by system A for their own activity.

Trp was the only amino acid for which there was a failure to concentrate in cord blood as compared to maternal, in the PM with IV group. In idiopathic FGR, Trp concentration occurs [45] implying that the failure of Trp to concentrate in our cases was related to the presence of PM rather than FGR *per se*. Other essential amino acids (Ile, Leu, Phe, Thr, Val), which, like Trp, are transported predominantly by system L [50], were concentrated in cord blood as compared to maternal, suggesting that there was no global impairment of system L activity. In placental infections, Trp is catabolised through the kynurenine pathway, notably by the enzyme indoleamine 2,3 dioxygenase [51], and we speculate that similarly increased placental catabolism of Trp in PM contributes to the lack of Trp placental concentrative capacity we observed. Cord Trp concentration was increased in PM without IV, suggesting that Trp placental catabolism may be reduced with acute infection; a change that is then blocked by inflammatory cells in chronic infection.

Taken together, our data suggest that PM alters placental function through effects on multiple amino acid transporter systems, and that these effects are selective for certain amino acids; PM may also increase placental amino acid metabolism. In PM with IV, the dysregulation of maternofetal amino acid concentrations is more pronounced, possibly because the monocytes accumulating in the intervillous space release inflammatory mediators that alter the activity of amino acid transporters in the syncytiotrophoblast.

We have shown effects of PM on one amino acid transport system, system A, *in vitro* and *ex vivo*, and found clues for the dysregulation of other placental amino acid transport systems. Amino acid transport is highly complex, with overlapping and interdependent pathways. Although the defects we observed in amino acid transporter activity did not translate directly into lower fetal amino acid concentrations in women with PM and IV, we did observe important correlations in women with PM, or PM and IV, between low fetal/placental weight ratio, an index of placental insufficiency, and low cord levels of critical amino acids. Our evidence calls for studies to further characterize the effects of PM and IV on the activity of system A as well as investigating the activities of systems X_AG_^−^ and L in the placenta. Such studies should also capture whether PM with IV alters transplacental transport of glucose [52] or lipids which, together with amino acids, form essential substrates for fetal growth [53], [54].

In order to counteract the decrease in placental nutrient transport, nutrient supplementation interventions [55] could be implemented, but should ideally be combined with further research to ensure that such interventions correct, and do not exacerbate [56], defects in transport and fetal growth [57].

Greater understanding of the mechanisms by which PM affects placental nutrient transport, combined with possible interventions to improve fetal growth in malaria, are important priorities in areas of the world where the co-existence of malaria and maternal malnutrition threaten the health and lives of millions of young babies.

## Materials and Methods

### Recruitment of participants

From 2001–2006, pregnant women delivering a live singleton newborn in the labor ward of Queen Elizabeth Central Hospital, Blantyre, Malawi were recruited into a case-control study. Cases were defined by the presence of *P. falciparum* asexual parasites on placental blood smear. For each case identified, two uninfected, age (±2 years) and gravidity-matched controls, negative for malaria parasites by both peripheral and placental smears, were then enrolled. Inclusion and exclusion criteria have been described elsewhere [41]. The College of Medicine Research Ethics Committee, University of Malawi, approved the study and written informed consent was obtained from all participants. Immediately after delivery maternal and cord venous blood were collected and separated by centrifugation. Plasma was stored at −80°C.

One set of placental biopsies was snap-frozen in liquid nitrogen for MVM purification, another set was embedded in optimal cutting temperature (OCT) medium before being frozen at −80°C for laser capture microdissection (LCM) of the syncytiotrophoblast and a last set was fixed in 10% neutral-buffered formalin for malaria infection grading. Placental tissue sections were examined by light microscopy for presence of malaria infection. In infected placentas, 500 randomly-selected intervillous space maternal blood cells were counted as previously described [5], to derive estimates of placental parasite density and monocyte counts (expressed as percentage of all maternal intervillous cells).

### Sample selection

Samples used in the study were selected from the cohort of participants based on tissue availability, after assessment of placental histology as described below. Presence of IE in the intervillous space of the placenta defined PM cases. These were sub-grouped into PM with or without IV. IV was defined as a monocyte count ≥5% of all intervillous cells counted [5]. Uninfected placentas were defined as showing no signs of malaria infection or intervillositis.

### Quantitative PCR analysis of *SLC38A1* and *SLC38A2* mRNA expression

RNA was extracted from laser-captured syncytiotrophoblast as previously described [58]. Briefly, tissue cryosections immobilized on SuperFrost PLUS slides (Fisher) were air-dried and fixed in acetone. After rehydration, sections were stained with methyl green (Sigma-Aldrich) and dehydrated. Material captured by laser microdissection using a MicroBeam microscope (P.A.L.M. Microlaser Technologies) was catapulted directly into RNA extraction buffer (RLT buffer with β-mercaptoethanol; Qiagen), and RNA extracted using an RNeasy Micro Kit (Qiagen), according to the supplier's recommendations. Purified RNA was eluted and kept at −80°C. RNA (10 ng) was reverse transcribed using Superscript III enzyme mix (Invitrogen) with random hexamers. Transcript levels for *SLC38A1* (SNAT1), *SLC38A2* (SNAT2) and tyrosine 3-monooxygenase/tryptophan 5-monooxygenase activation protein, zeta polypeptide (*YWHAZ*) were quantified using 1∶4 dilution of cDNA for all samples. Primer sequences are shown in Table 4. Real-time quantitative PCR was performed using previously reported thermal cycling conditions at an annealing temperature of 60°C with SYBR Green 1 (Applied Biosystems) [59]. Transcript levels were quantified against a standard curve generated from a pool of all placental cDNA samples. Preliminary studies confirmed *YWHAZ* transcript levels were comparable between groups and *YWHAZ* was used to normalize target gene transcript levels.

**Table 4:** Primer sequences used for qPCR.

Target Gene	Forward primer	Reverse primer
*SLC38A1*	AAGAAACAGGCTGCATGGTGTA	CCCTGTGGTGCCAAAGACTT
*SLC38A2*	TTCAGTTGGTGGCGTCATAGT	AATCAACATAGAAGCTGCAGATGC
*YWHAZ*	ACTTTTGGTACATTGTGGCTTCAA	CCGCCAGGACAAACCAGTAT

All sequences are 5′-3′.

### MVM vesicle isolation

Isolation of MVM vesicles from placental biopsies (7.2±1.6 g) was performed using magnesium precipitation and differential centrifugation based on the method of Glazier *et al.* [60] as described previously [61] with modifications according to Jimenez *et al.* [62] to allow simultaneous recovery of the basal plasma membrane for other studies.

Frozen placental biopsies were thawed and homogenized in 250 mM sucrose, 10 mM HEPES-Tris, pH 6.95 (Buffer D; 2.5 volumes biopsy weight) and a sample of the homogenate (1 ml) was retained for further analysis. The remaining homogenate was centrifuged at 10000 *g* for 15 min at 4°C and the supernatant retained. The pellet was resuspended in buffer D (1.5 volumes of initial biopsy weight) and the centrifugation step was repeated. The supernatants were pooled and centrifuged at 125000 *g* for 30 min at 4°C. The pellet was resuspended in buffer D and 12 mM MgCl_2_ added and stirred on ice for 20 min. The suspension was centrifuged at 2500 *g* for 10 min at 4°C. The supernatant (containing MVM) was centrifuged at 125000 *g* for 30 min at 4°C. The pellet was resuspended in 300 mM sucrose, 20 mM Tris-maleate, pH 7.4 and loaded onto a discontinuous 25%–37%–45% sucrose gradient. After centrifugation at 90000 *g* for 6 h at 4°C, the MVM fraction at the 37%–45% interface was recovered and centrifuged at 110000 *g* for 30 min at 4°C. The resultant MVM pellet was resuspended in intravesicular buffer (290 mM sucrose, 5 mM HEPES, 5 mM Tris-HCl, pH 7.4; 3 volumes pellet weight) and repeatedly passed through a 25-gauge needle to vesiculate the MVM fragments to form vesicles. MVM vesicles were stored at −80°C.

Protein concentration of placental homogenate and MVM vesicles was determined by the Lowry method [63]. Purity of MVM vesicle preparations was assessed by enrichment of alkaline phosphatase activity as described previously [60]. Alkaline phosphatase enrichment factors (mean ± SD) were not different (p = 0.39) between uninfected (16.8±8.6; n = 18), PM (11.5±6.0; n = 14) and PM with IV (12.9±6.7; n = 21) groups, suggesting comparable MVM purity between groups.

### ^14^C-MeAIB uptake

^14^C-MeAIB (NEC-671; PerkinElmer,) was used as a well-characterized, non-metabolizable amino acid analogue substrate to measure the activity of system A amino acid transporter [15], [17].

#### MVM vesicles

^14^C-MeAIB uptake by MVM vesicles was measured as described previously in the presence and absence of an inwardly-directed Na^+^ gradient with the Na^+^-dependent component taken to reflect system A-mediated MeAIB uptake [17]. Uptake was initiated by the addition of 20 µl MVM vesicle suspension (∼430 µg protein) to 20 µl extravesicular buffer (5 mM HEPES, 5 mM Tris, 145 mM NaCl or 145 mM KCl, 0.165 mM ^14^C-MeAIB, pH 7.4). Na^+^-dependent uptake of ^14^C-MeAIB into MVM vesicles at 30 s was taken to provide an estimate of initial rate, in accordance with previous studies [17], [19].

#### BeWo cells

The BeWo choriocarcinoma trophoblast cell line (kindly provided by Prof Robin Mortimer, Placental Transport Unit, Queensland Institute of Medical Research, Australia) was cultured in DMEM/Ham's F12 medium, supplemented with 10% fetal bovine serum and penicillin/streptomycin (all from Gibco). The cells were passaged every 4–7 days and passages 12–30 were used. Cells were seeded in 24-well culture plates at 2.5×10^5^ cells/well in 0.5 ml culture medium for 24 h at 37°C before the culture medium was replaced by the stimuli. After 16 h, supernatants were aspirated, and cells were washed with 0.5 ml transport buffer (25 mM HEPES, 25 mM Tris, 140 mM NaCl, 5.4 mM KCl, 1.8 mM CaCl_2_, 0.8 mM MgSO_4_ and 5 mM glucose, pH 7.5) at 37°C. Then, 0.25 ml transport buffer containing 0.25 µCi ^14^C-MeAIB was added to each well for 20 min in 5% CO_2_ in air at 37°C. In parallel, transport of MeAIB by Na^+^-independent pathways was measured, using a modified transport buffer, in which KCl was substituted for NaCl. Transport buffer was aspirated and cells washed thrice with ice-cold transport buffer and lysed in 0.6 ml 1% SDS in 0.2 M NaOH. Lysates were stored at −20°C until the radioactivity was quantified by liquid scintillation counting in 5 ml Optiphase Hisafe 2 (PerkinElmer) and the lysate protein concentration was measured by Lowry assay. In parallel, the impact of stimuli on cell viability was assessed by using the Alamar Blue assay (Invitrogen) according to the manufacturer's instructions [64].

### IL-1β ELISA

Placental blood was aspirated from an incision made in the basal plate at a pericentral site of the placenta. Plasma was separated and frozen at −80°C. IL-1β was measured by ELISA (DuoSet, R&D) in undiluted plasma samples according to the manufacturer's instructions.

### Monocyte/IE co-culture

#### Monocyte isolation and *P. falciparum* culture

Buffy coats from volunteer blood donors (Australian Red Cross Blood Services) were used to isolate CD14^+^ cells by positive immunoselection using anti-CD14 magnetic beads (Miltenyi Biotec). The purity of the CD14^+^ cells as assessed by flow cytometry was >95%.

*P. falciparum* blood stage parasites (CS2 line) were maintained in O^+^ erythrocytes (Australian Red Cross Blood Services) at 2–5% hematocrit in RPMI-HEPES medium supplemented with 0.5% Albumax II (Gibco) and 25 mM NaHCO_3_ at 37°C in 1% O_2_/5% CO_2_/94% N_2_. Cultures were regularly synchronized by sorbitol treatment [65] and knob-expressing IE were selected by sedimentation on gelatin every 2 weeks [66]. No *Mycoplasma* contamination of the cultures was detected by PCR.

#### Co-culture

CD14^+^ cells were plated out in 24-well tissue culture-treated plates (Falcon BD biosciences) at 5×10^5^ cells/well (0.5 ml/well in DMEM/Ham's F12, 10% FBS with penicillin/streptomycin). Cells were incubated at 37°C in 5% CO_2_ in air for 1 h before 0.5 ml gelatin-purified IE were added (20 IE per CD14^+^ cell). An equal number of uninfected erythrocytes (UE) were added to control wells. After 24 h, supernatants were collected, spun at 2000 *g* for 5 min and cell-free supernatants stored at −80°C.

### Measurement of free amino acids

Samples from primigravidae were preferentially selected for the amino acid analysis, as they are at highest risk of malaria in pregnancy [2]. In paired maternal and cord blood plasma samples, free forms of the common 20 amino acids were analyzed by reversed phase ultra performance liquid chromatography (RP-UPLC) with pre-column derivatization with 6-aminoquinolyl-N-hydroxysuccinimidyl carbamate (AccQ Tag Ultra; Waters Corporation). Standards (Amino Acid Standard H (Pierce) together with Gln, Trp and Asn (Sigma)) were prepared with norvaline (Sigma) included as an internal standard. For assay, 100 µl plasma was mixed with an equal volume of 200 µM norvaline and deproteinated by ultrafiltration through a 10 kDa MWCO spin filter (Millipore) at 4800 *g* for 60 min at 10°C. 10 µl filtrate was derivatized and analyzed on a Waters Acquity UPLC over a 20 min gradient using a 2.1×150 mm, 1.7 µm i.d., BEH C18 column (Waters Corporation) flowing at 0.6 ml/min at 60°C. Detection was via UV (260 nm) and data was collected and analyzed using the Waters Empower2 software.

### Statistical analysis

Non-normally distributed data are presented as box plots showing the median, 25^th^/75^th^ and 10^th^/90^th^ centiles unless described otherwise. Values out of the 10^th^ centiles were included in statistical analyses but not represented in graphs. Normally distributed values are presented as mean and standard deviation. Non-normally distributed data were normalized by log-transformation prior to statistical analysis. Data were then compared between 3 groups using one-way ANOVA. When the p value of the ANOVA test was lower than 0.1, two-group comparisons were made using a 2-tailed T-test. Correlations were assessed using Pearson's correlation test. Trends across ordered groups were tested using Cuzick's test. The ability of the placenta to concentrate amino acids in cord blood was examined by testing if the cord to maternal concentration ratio of each amino acid was different from 1 using a one-sample T-test.

### Study approval

The College of Medicine Research Ethics Committee, University of Malawi, approved the study and written informed consent was obtained from all participants prior to inclusion in the study.
